# Optimizing Rhinoplasty Outcomes: The Role of Isotretinoin and Patient Management Strategies: Systematic Review

**DOI:** 10.1111/jocd.70575

**Published:** 2025-11-27

**Authors:** Matin Ghazizadeh, Behrouz Barati, Ali Goljanian Tabrizi, Mohsen Jabbari Joushin, Sina Hooshmandinia

**Affiliations:** ^1^ Associate Professor of Otorhinolaryngology, Department of Otorhinolaryngology Shahid Beheshti University of Medical Sciences Tehran Iran; ^2^ School of Medicine Shahid Beheshti University of Medical Sciences Tehran Iran

**Keywords:** aesthetic outcomes, isotretinoin, postoperative recovery, rhinoplasty, thick nasal skin, wound healing

## Abstract

**Background:**

Rhinoplasty outcomes in patients with thick nasal skin may be challenging due to the sebaceous nature of the skin, which can compromise tip definition and delay resolution of postoperative edema. Perioperative adjunct using isotretinoin is suggested as a well‐established treatment for acne and sebaceous hyperactivity.

**Methods:**

We conducted this systematic review in accordance with PRISMA guidelines. Literature searches were performed in PubMed, Scopus, Web of Science, and Embase databases for studies published up to March 2025. Search strategies incorporated combinations of keywords relating to “rhinoplasty,” “isotretinoin,” “nasal skin,” and “wound healing.” Eligible studies included randomized controlled trials (RCTs), cohort and retrospective studies, with human participants undergoing rhinoplasty with or without systemic or topical isotretinoin, reporting on outcomes such as skin thickness, edema, patient satisfaction, wound healing, and complications. Risk of bias and quality assessment were assessed with the Joanna Briggs Institute (JBI) critical appraisal tools.

**Results:**

This systematic review included seven eligible studies—all with human participants—three RCTs, three retrospective cohort studies, and one prospective cohort study. We found that isotretinoin improved nasal tip definition, decreased postoperative edema, and improved patient satisfaction. Specific studies reported a marked reduction in sebaceous gland activity, and shaved‐off nasal skin thickness was considered responsible for the more refined aesthetic outcome. No significant adverse effects other than mild mucosal dryness and transient cheilitis were observed; it seemed to accelerate wound healing. However, causality within isotretinoin use could not be established in some studies, and some needed revision surgery.

**Conclusion:**

Isotretinoin administration, particularly when individualized by patient profile, appears to significantly improve both aesthetic and wound‐healing outcomes in rhinoplasty patients with thick nasal skin and displays a favorable safety profile under close monitoring. Further large‐scale RCTs are required to optimize dosage and perioperative protocols.

## 1 Introduction

Rhinoplasty has become rather globally popular. As a result, rhinoplasty is one of the top global cosmetic surgeries. The 2020 Plastic Surgery Statistics Report states that rhinoplasty (or nose surgery) earned $1.9 billion in 1 year, confirming that this came from 352 555 procedures. Of those, 81.5% are females, while 67.74% are individuals aged 13–40, when the sensitivity of looks is higher than usual [1]. Therefore, performing rhinoplasty in people with thick nasal skin is problematic because it is difficult to achieve a contoured, ideal tip definition throughout the procedure [2]. This is especially true for those of African or Middle Eastern heritage who have generally weak nasal cartilages and thick dermis [3].

Continuing the trend of recognizing the importance of skin thickness and subcutaneous soft tissues, facial cosmetic procedures are increasingly considered to involve these attributes. Additionally, acne is more common in those with a dense skin soft‐tissue envelope and in those seeking rhinoplasty only, that is, those seeking cosmetic treatment for aesthetic reasons (i.e., teenagers and young adults) [4]. Patients with thick nasal skin are at higher risk of less favorable rhinoplasty outcomes, including a blurred nasal tip and supratip pathology. The patients should be preassessed and categorized. Almost all of the various categories are the same thing [5]. According to Cobo et al. [6], they eliminated individuals with thin nasal skin and classified persons with thick skin into three types. It assumed such a categorization based on expected dermal attributes before and after the procedure (dorsal, cleaned and unclean, and damaged and undamaged) and presurgical (emperor, carpus, incised, incision edges, far carpus) as well as postsurgical (surgical and nonsurgical treatments; swelling, necrosis, hematoma, reabsorption, granulation tissue, scarring, mal‐dependency) interventions [6]. Treatment strategies tailored to each skin type, including topical and surgical approaches that may begin and continue for many months before and after the operation, are reported. The regulation of skin thickness due to excessive sebaceous activity is proposed to be achieved through an appropriate diet and Retin A and isotretinoin therapy [5, 7].

It has been used over the years to treat rhinophyma and acne using isotretinoin. Others have investigated whether oral isotretinoin may improve tip definition in patients with thick skin [8]. In thick‐nasal‐skin patients, Guyuron and Lee [9] systematically formulated a management protocol for rhinoplasty patients and stressed the importance of considering the skin phase as a key [9]. For patients with sebaceous hyperactivity, topical Retin‐A was recommended as essential. Researchers investigate whether the fewer side effects of topical tretinoin may be used for the management of thick‐skinned rhinoplasty, as oral isotretinoin may have a considerable impact on the patient's systemic system [7].

Although previous reviews [5, 6] and narrative reports have documented oral isotretinoin's potential in postrhinoplasty management, major gaps remain: no systematic review to date has comprehensively compared both oral and topical isotretinoin interventions nor synthesized findings across diverse populations and outcome domains (skin thickness, wound healing, patient satisfaction). Therefore, the present systematic review aims to fill these gaps by [1] incorporating a broader range of isotretinoin interventions and patient management strategies for thick‐skinned rhinoplasty patients [2], providing a transparent, reproducible methodology, and [3], advancing understanding of both efficacy and safety parameters based on current human clinical evidence.

## 2 Methods

### 2.1 Study Design

**FIGURE 1:**
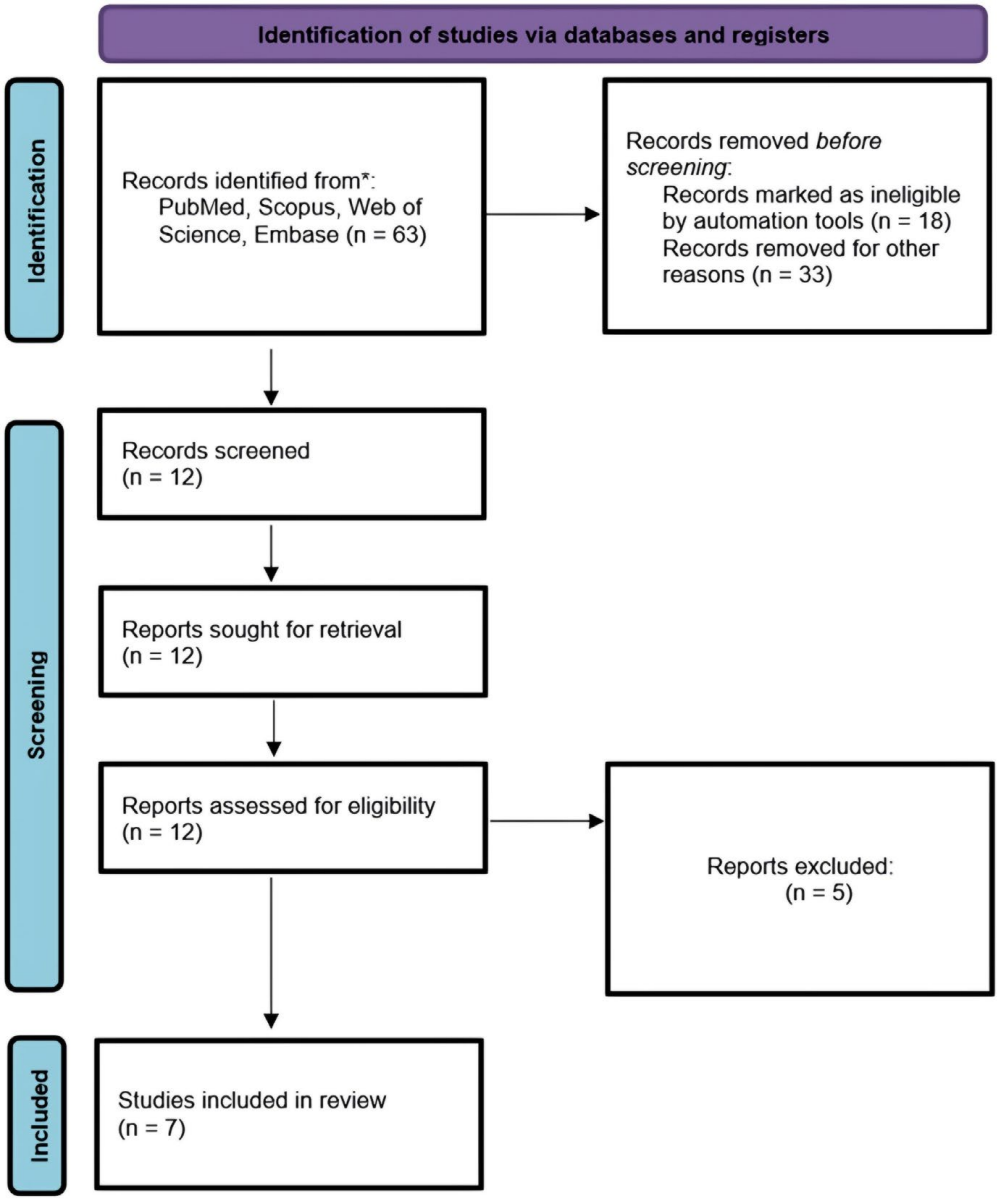
The flow diagram of the study.

It is a systematic review of the effect of isotretinoin on rhinoplasty outcomes in patients with thick nasal skin. To maintain transparency and methodological rigor, the review is also conducted according to PRISMA (Preferred Reporting Items for Systematic Reviews and Meta‐Analyses) guidelines [10]. A strategy for a structured search of relevant databases, data extraction, and synthesis was conducted systematically to identify studies registered as eligible (Figure 1).

### 2.2 Search Strategy

Our comprehensive search strategy covered PubMed, Scopus, Web of Science, and Embase databases to find studies published up to March 2025. We used a variety of keyword combinations as search terms to ensure that no relevant studies were overlooked.

- “Rhinoplasty” OR “Nasal surgery”
- “Isotretinoin” OR “Retinoids” OR “Topical tretinoin”
- “Thick nasal skin” OR “Sebaceous skin” OR “Acne‐prone skin”
- “Dermatologic outcomes”; “Wound healing”; “Patient satisfaction”

The search strategy was refined using Boolean operators (AND, OR). In addition, the reference lists of included studies and relevant systematic reviews were screened for additional studies (Table 1).

**TABLE 1:** Search strategy of this systematic review.

Database	Search string/Query used	Search date
PubMed	(“rhinoplasty”[MeSH] OR “rhinoplasty”[tiab] OR “nasal surgery”[tiab]) AND (“isotretinoin”[MeSH] OR “isotretinoin”[tiab] OR “retinoids”[tiab] OR “tretinoin”[tiab]) AND (“thick nasal skin”[tiab] OR “sebaceous skin”[tiab] OR “acne prone skin”[tiab])	Mar 2025
Scopus	TITLE‐ABS‐KEY (rhinoplasty OR “nasal surgery”) AND TITLE‐ABS‐KEY (isotretinoin OR retinoids OR tretinoin) AND TITLE‐ABS‐KEY (“thick nasal skin” OR sebaceous OR acne)	Mar 2025
Web of Science	TS = (rhinoplasty OR “nasal surgery”) AND TS = (isotretinoin OR retinoids OR tretinoin) AND TS = (“thick nasal skin” OR sebaceous OR acne‐prone)	Mar 2025
Embase	(‘rhinoplasty’/exp. OR ‘rhinoplasty’:ti, ab OR ‘nasal surgery’:ti, ab) AND (‘isotretinoin’/exp. OR ‘isotretinoin’:ti, ab OR ‘retinoid’/exp. OR ‘tretinoin’:ti, ab) AND (‘thick nasal skin’:ti, ab OR sebaceous:ti, ab OR acne:ti, ab)	Mar 2025

Abbreviations: Exp, explode; Tiab, title/abstract; TS, topic search, includes all sub‐terms under MeSH/Emtree.

### 2.3 Eligibility Criteria

Our inclusion criteria were stringent, ensuring that only studies meeting the following criteria were included in our review.

1. Study group: Patients having rhinoplasty, emphasizing patients with thick or sebaceous nasal skin.
2. Intervention: Use of systemic or topical isotretinoin before or after surgery.
3. Standard care alone (if applicable), if any, compared to control groups receiving standard care without isotretinoin.
4. Aesthetic outcomes, patient satisfaction, wound healing, postoperative complications, or histological changes in skin thickness.
5. Randomized controlled trials (RCTs), cohort studies, retrospective studies, and experimental studies.

The exclusion criteria were case reports, non‐English publications, animal studies unless directly applicable to human applications, and studies with unclear endpoint measurements.

### 2.4 Study Selection

The study selection process was transparent and unbiased. After retrieving the studies, two independent reviewers screened them based on titles and abstracts. Full‐text articles of potentially relevant studies were then included for eligibility, and any discrepancies in the study selection were discussed with a third reviewer.

### 2.5 Data Extraction

Information from each included study was extracted using a standardized data extraction form. Extracted data included:

- Study characteristics (author, year, country, study design)
- Sample size, age, gender, skin type classification (patient demographics)
- The interval of timing relative to surgery, duration, and total intervention dosage.
- Outcome measures included aesthetic improvement, patient satisfaction, nasal skin remodeling, wound healing, and complications.
- Follow‐up duration and key findings

### 2.6 Risk of Bias and Quality Assessment

The methodological quality and risk of bias for all included studies were assessed using the Joanna Briggs Institute (JBI) critical appraisal tools. The JBI checklist for RCT was applied to interventional studies, and the JBI checklist for cohort studies was used for observational (cohort and retrospective) studies. These tools evaluate key domains, including selection bias, confounding, data collection, outcome measurement, and reporting quality. Two reviewers independently appraised each study, and any disagreements were resolved through consensus.

### 2.7 Data Synthesis and Analysis

This was followed by a qualitative synthesis of the findings summarizing the effect of isotretinoin on rhinoplasty outcomes. A meta‐analysis was only applied if sufficient homogeneous data were available due to heterogeneity in study designs, interventions, and outcome measures. If a meta‐analysis was conducted, statistical heterogeneity was first assessed using the I^2^ statistic, and the effect sizes were reported as MD or with 95% CI.

### 2.8 Ethical Considerations

Ethical approval was not necessary since this is a systematic review of previously published research. Nevertheless, all included studies were verified to follow ethical guidelines, including obtaining informed consent from participants and obtaining approval from institutional review boards, where available.

## 3 Results

Seven studies meeting the inclusion criteria for this systematic review evaluated the role of isotretinoin in achieving the ideal rhinoplasty outcomes. These were retrospective or prospective studies and RCTs that examined aesthetic outcomes, wound healing, postoperative complications, and patient satisfaction (Table 2).

**TABLE 2:** Basic characteristics of included studies.

Author year country	Study design and time frame	*N* (Groups)	Age (Years)	Women, *n* (%)	Intervention	Fitzpatrick scale nasal skin type, *n*	Isotretinoin treatment protocol	Reported outcomes	Reported postoperative time period (mean, SD, range, months)	Complications
Baser, Dev et al. 2024, India [11]	Retrospective	26	20–35	18 (69%)	Rhinoplasty Type: Primary [12] & Secondary [3]	Type NS. 26 reported to have thick acne‐prone skin	Treatment initiated 4–6 weeks preop:—Treatment: Combined topical+systemic—Topical: Daily salicylic acid/benzoyl peroxide wash, tretinoin cream (0.025%) at bedtime—Systemic: Oral isotretinoin 20 mg daily—Topical treatment stopped 5 days preop, resumed postop day 10—Systemic treatment stopped 1 week preop, resumed 2–3 weeks postop—Combined regimen continued 6–8 months postop—Postop: Triamcinolone (10 mg/mL) nasal supratip every 6 weeks until edema resolved	Pre/− v Postoperative assessment: (1) ROE (2) Subjective patient satisfaction (3) Image	5.5 (4.8), 1–12	Few patients: Mid‐cheilitis 2 patients: Dry skin, cheilitis (Treatment dose reduced) 1 patient: Elevated liver enzymes (Treatment discontinued)
Cobo and Vitery 2016, Colombia [1]	Retrospective 2010–2015	17	—	10 (60%)	Rhinoplasty Type: NR	Type NS. 17 with thick acne‐prone skin, bulbous, undefined nasal tips	Treatment initiated at 1 month postop:—Treatment: Oral isotretinoin (0.25–0.5 mg/kg) for 4–6 months	Pre/− v Postoperative assessment: (1) Patient Image	15.8 (14.2), 1–36	Dry skin, eyes, lips, intranasal mucosa (treated with lubricants)
Pozzi, Fàdel et al. 2023, Italy [13]	Retrospective 01/2019–10/2022	1321	17–63	92%	Rhinoplasty—Primary only	Type NS. 1321 non‐Caucasian patients	Treatment initiated at 1 week postop:—Treatment: Oral isotretinoin (dose NS) for 3 months—Postop: Subdermal corticosteroid injections for persistent inflammation/edema	Pre/− v Postoperative assessment: (1) ROE (2) Visual analog scale for pain (3) Patient image	14 (9.2), 6–24	39 patients: Minor infections 3 patients: Skin necrosis (treated in hyperbaric room)
Sazgar, Majlesi et al. 2019, Iran [8]	Prospective—RCT 2014–2016	48 (24 isotretinoin, 24 placebo)	17–45	39 (81.3%)	Rhinoplasty—Primary only	Type NS. 48 with thick skin	Treatment initiated at 31 days postop:—Treatment: Oral Isotretinoin (0.5 mg/kg) ‐ Every other day for 1 month, daily for 2 months ‐ Continued for 3 months postop	Pre/− v Postoperative assessment: (1) Image (2) 5‐point aesthetic outcomes grading scale (surgeon) (3) 5‐point aesthetic satisfaction grading scale (patient)	7 (4.6), 3–12	Nasal dryness (2 patients) Bloody nasal discharge (1 patient)
Silveira, Azulay‐Abulafia et al. 2024, Brazil [14]	Prospective—RCT	24 (12 isotretinoin, 12 placebo)	18–45	24 (100%)	Rhinoplasty Type: NR	Type III [13], Type IV [7], Type V [5]	Treatment initiated at 2 months preop: ‐ Treatment: Oral Isotretinoin (0.25–0.4 mg/kg/day) for 4 months ‐ Stopped 15 days preop, restarted 3–5 days postop	Pre/− v Postoperative assessment: (1) Ultrasonography assessment of epidermis and dermis (2) Utrecht Questionnaire	NR	12 patients: Labial mucosa dryness 1 patient: Elevated triglycerides (resolved posttreatment)
Yahyavi, Jahandideh et al. 2020, Iran [15]	Prospective—RCT 2012–2015	350 (175 isotretinoin, 175 placebo, 303 completed)	18.7–39.2	215 (61%)	Rhinoplasty Type: NR	Type NS. Thick oily skin, large fleshy nose tips, with/without acne	Treatment initiated at 2 weeks preop: ‐ Treatment: Oral Isotretinoin 20 mg daily (0.3 mg/kg/day) ‐ Treatment continued for 2 months postop	Pre/− v Post assessment: (1) Image (surgeon) (2) Facial skin oiliness on a scale of 1–5 (Surgeon, Dermatologist) (3) Facial acne severity on a scale of 1–5 (Surgeon, Dermatologist) (4) Patient Satisfaction of nasal appearance on a Likert scale of 1–10 (Surgeon, Dermatologist) (5) Qualitative assessment: Deformities of nasal cartilages, Keloid tissue formation, scar healing (Surgeon, Dermatologist)	5.5 (4.8), 1–12	No reported complications
Yigit et al. 2022, Turkey [4]	Prospective study	40	Mean 20.9 ± 3.0	26 (65%)	Oral isotretinoin at 0.25 mg/kg/day or 0.5 mg/kg/day	Acne vulgaris patients	0.25 mg/kg/day or 0.5 mg/kg/day for 4 months	Significant decrease in dermis and subcutaneous tissue thickness at nasal landmarks; increased elasticity	4 months	—

Abbreviations: NR, not reported; NS, not specified; Postop, postoperative; Preop, preoperative.

### 3.1 Aesthetic Outcomes and Patient Satisfaction

The patients were divided into two subgroups based on the grade of acne and then compared to see whether they had thick, oily, or acne‐prone skin, and the effect of isotretinoin on nasal aesthetics and patient satisfaction.

Baser et al. [11], retrospectively studied 26 patients undergoing the first and second rhinoplasty. A combined oral and topical isotretinoin regimen consisting of 20 mg daily oral and topical tretinoin cream was used. 76.9% of patients reported high satisfaction at 1 year; although there was no control group, the improvement in ROE scores was significantly greater than baseline.

Pozzi et al. [13], also studied 1321 non‐Caucasian patients who underwent primary rhinoplasty and received isotretinoin 1 week postoperatively, with continued treatment for 3 months; isotretinoin significantly improved nasal definition and enhanced patient satisfaction.

In Sazgar et al. [8], 48 patients participated in an RCT; oral isotretinoin (0.5 mg/kg) was initiated 1 month after surgery and continued for 3 months. It was observed that scores on the aesthetic outcomes grading scale were higher in the isotretinoin group. The rate of ‘excellent’ satisfaction at 3 months was 41.6% (isotretinoin) versus 16.6% (placebo), *p* < 0.05; at months: 62.5% versus 33.3%, *p* < 0.05; and at 12 months: 75% versus 66.7%, *p* > 0.05.

Yahyavi et al. [15], also conducted an RCT with 350 patients using oral isotretinoin, initiated 2 weeks before surgery and continued for 2 months afterward. At 1, 3, 6, and 12 months postoperatively, patients in the isotretinoin group reported better nasal tip definition, less oiliness, and greater satisfaction than those in the control group. The mean satisfaction score in the experimental (isotretinoin) group at 1 and 3 months was significantly higher than in the control group (*p* < 0.01); at 6 and 12 months, scores remained slightly higher, but the difference was not statistically significant (*p* > 0.05).

### 3.2 Skin and Soft Tissue Remodeling Impact

Results from studies investigating changes in nasal skin thickness and soft tissue during the isotretinoin timeline found that isotretinoin thinned nasal skin and improved its elastic properties.

In two RCTs, oral isotretinoin (20 mg per day) was administered before and after rhinoplasty, according to Silveira et al. [14]; epidermal and dermal thickness were significantly reduced at the nasal dorsum and wing. Cobo and Vitery [1], in a study of 17 patients with thick, acne‐induced skin, found that therapeutic doses of isotretinoin (4–6 monthly) administered for 1 month postoperatively result in fewer cases of postoperative swelling and more defined nasal tip contours.

Yigit et al. [4] conducted a prospective study on acne vulgaris patients. In 40 acne patients, isotretinoin (0.25 or 0.5 mg/kg/day) led to a ~26% mean reduction in dermis thickness at the nasal tip over 4 months (pre = 1.4 ± 0.5 mm; 4th month = 0.9 ± 0.3 mm; *p* = 0.001). Supratip region: ~36% reduction (1.4 ± 0.6 mm to 0.9 ± 0.3 mm, *p* = 0.001). Nasion: 0.80 ± 0.3 mm to 0.50 ± 0.1 mm (*p* = 0.001). Subcutaneous soft tissue thickness: tip from 2.0 ± 0.6 to 1.5 ± 0.5 mm (*p* = 0.001); supratip 2.2 ± 0.6 to 1.5 ± 0.4 (*p* = 0.001). No significant difference between 0.25 and 0.5 mg/kg groups (*p* > 0.05).

### 3.3 Wound Healing and Scar Formation

Across included studies, isotretinoin demonstrated a positive effect on wound healing and skin remodeling after rhinoplasty in patients with thick nasal skin, with accelerated resolution of postoperative edema and no evidence of delayed wound healing in monitored human subjects.

### 3.4 Postoperative Complications and Safety

The mild and manageable adverse effects associated with isotretinoin. Complications most commonly reported were dry skin, cheilitis (lip dryness), and nasal dryness. In Sazgar et al. [8], at 3 and 6 months post‐op, improvements in surgeon‐rated cosmetic results and reductions in edema were significantly greater with isotretinoin than with placebo (*p* < 0.05). By 12 months, the difference was no longer statistically significant (*p* > 0.05). Furthermore, according to Baser et al. [11], one patient developed mid‐cheilitis as well as elevated liver enzymes, which required dose adjustments. In dry eyes, lips, and intranasal mucosa, lubricants were noted by Cobo and Vitery [1], and they were successfully treated. Twelve patients presented with mild mucosal dryness, as Silveira et al. [14] reported, which resolved upon discontinuation of isotretinoin.

However, according to Yahyavi et al. [15], there was no difference in delayed wound healing, abnormal scarring, keloid formation, or cartilage deformities between groups (*p* > 0.05). Adverse events were minor: cheilitis, dermatitis. Yigit et al. [4], reported that laboratory findings remained within the normal range at the end of follow‐up in the isotretinoin group. All studies emphasized strict monitoring for teratogenicity and metabolic effects. However, most importantly, no study was reported to have had severe complications, including hypertrophic scarring, delayed wound healing, or systemic toxicity.

### 3.5 Risk of Bias and Quality Assessment

Among the seven included studies, three RCTs and four cohort studies were evaluated using the appropriate JBI appraisal tool. Most studies demonstrated moderate to high methodological quality, with low risk of bias in recruitment, exposure measurement, and outcome reporting. Full details of the appraisal for each study are provided in (Table 3).

**TABLE 3:** Risk of bias and methodological quality assessment of included studies based on the Joanna Briggs Institute (JBI) tools.

Study (Author/Year)	Study design	Tool (JBI checklist)	JBI score	Overall JBI rating
Silveira et al. 2024	RCT	JBI RCT	12/13	Low risk
Sazgar et al. 2019	RCT	JBI RCT	12/13	Low risk
Yahyavi et al. 2020	RCT	JBI RCT	11/13	Moderate risk
Baser et al. 2024	Cohort	JBI Cohort	10/11	Low risk
Cobo & Vitery 2016	Cohort	JBI Cohort	9/11	Moderate risk
Pozzi et al. 2023	Cohort	JBI Cohort	11/11	Low risk
Yigit et al. 2022	Cohort	JBI Cohort	9/11	Moderate risk

## 4 Discussion

This systematic review suggests that isotretinoin improves rhinoplasty outcomes, especially in patients with thick or oily skin. These results demonstrate significant improvements in aesthetic outcomes, patient satisfaction, skin remodeling, wound healing, and postoperative edema.

### 4.1 Wound Healing and Postoperative Recovery

Baser et al. [11], and Cobo and Vitery [1], reported that, in clinical studies, postoperative edema resolved more quickly and the nasal tip was refined better with isotretinoin. This indicates that healing may be better with isotretinoin, and the swelling is less prolonged.

### 4.2 Isotretinoin: Mechanism and Effects

These retinoid agents include isotretinoin (13, 13‐cis retinoic acid), which is also used to treat severe and recalcitrant nodular acne [16, 17]. The FDA‐approved cell cycle, the protein, and the regulations were now on protein, on formulations, and on many effects on differentiation, life, neutrophil‐gelatinase‐associated, across size, and the life globe [1, 16, 17, 18, 19]. Sebaceous glands reduce these glands, increase sebum production under the direct influence of C. acnes growth and the microenvironment of the sebaceous ducts, and inhibit inflammation [20].

Aside from treating nodular acne, isotretinoin has been shown to treat conditions such as rhinophyma, sebaceous hyperplasia, and acne that do not respond to conventional therapy [21, 22]. It also increases dermcidin levels, an antimicrobial peptide that augments the skin's natural immunity, reducing inflammation and bacterial colonization. Isotretinoin is very effective, but its side effects, in particular, teratogenic effects, render strict monitoring of the prescription of the drug to avoid pregnancy [12, 19].

According to its pharmacokinetics, it has a mean half‐life of 20–39 h, and it is readily absorbed when given with a high‐fat (food) meal. Most of the time, after a great deal of liver metabolism (and the creation of a host of active metabolites), it is eliminated via feces. Isotretinoin, however, is limited by its potential side effects, especially its teratogenic risks [19, 23, 24], which limit its therapeutic benefits. As the drug is contraindicated for pregnant women and those who may become pregnant, safety precautions such as the US's iPLEDGE program (viii) attempt to ensure fetal exposure is limited [25].

This new research suggests using isotretinoin to treat other dermatological disorders, such as hidradenitis suppurativa, rosacea, some forms of cutaneous neoplasms, and acne [19]. The usual treatment dosages range from 0.1 to 2 mg/kg/day [19]. It helps to understand the complex mechanisms of isotretinoin and how it is used in other dermatology research, while discussing the importance of the drug in treating skin conditions, especially in patients undergoing surgery, such as rhinoplasty, who have thick skin or acne [18, 26].

### 4.3 Isotretinoin in Rhinoplasty

An approach to rhinoplasty, particularly for people of African descent, was investigated by Patrocinio et al. [27]. The use of oral isotretinoin and triamcinolone injection contributed to a better definition of the nasal tip [27]. A RCT using ultrasonography was conducted by Silveira et al. [14] to assess the impact of oral isotretinoin on patients undergoing rhinoplasty. There were 24 subjects, and the influence was determined preoperatively and postoperatively. The findings showed that isotretinoin reduced the thickness of the skin in the areas of the nasal regions tested.

In the 1980s, consideration was given to postponing elective treatments (such as dermabrasion, rhinoplasty, and laser therapy) for 6 months for those identified as being prescribed isotretinoin [28, 29].

Using a porcine model, Larson et al. [30] investigated healed partial‐ and full‐thickness wounds postsystemic isotretinoin treatment. Their findings show that invasive surgical procedures can be safely performed with recent isotretinoin therapy. Second, their results also question the conventional approach of waiting more than 6 months after completing isotretinoin treatment before surgery. Additionally, a retrospective study by Allen et al. [31], was conducted to assess the relationship between isotretinoin use during the postoperative period and the development of nasal tip deformities after rhinoplasty. Three cases of patients who underwent isotretinoin therapy following rhinoplasty were studied. All three patients ultimately underwent additional surgical intervention for soft‐tissue deformities of the nasal tip. Isotretinoin was initiated after 2 years of the initial rhinoplasty in each case, with the first appearance of nasal tip deformity within 6 months of isotretinoin treatment [31].

As part of the McDonald [32], review study, several publications from 2017 that question the need for a 6 month delay for all cosmetic procedures have been examined, and a shift in the established best practice is being proposed. However, the authors point out that the evidence is insufficient to support such a delay for many of them. According to their findings, superficial chemical peels, focal and manual superficial dermabrasion, fractional ablative or nonablative lasers, and laser hair removal may proceed without a 6 month delay. On the other hand, a 6 month delay should continue to be observed for full‐face dermabrasion, mechanical dermabrasion, and fully ablative treatments [32].

A RCT to assess the cosmetic outcome of rhinoplasty after the use of oral isotretinoin for thick nasal skin was conducted by Sazgar et al. [8]. A total of 48 participants were randomized to go on a double‐masked placebo‐controlled clinical trial. Moreover, oral isotretinoin (0.5 mg/kg) was initiated on the 31st day after surgery, administered every other day for 1 month, followed by daily for the remaining 2 months in the experimental group. Patients in the isotretinoin group also showed higher patient satisfaction and expert surgeon evaluation early (3 months after surgery) and late (6 months after surgery) than those in the placebo group. Nevertheless, no significant statistical difference between the two groups was observed at 12 months following surgery [8].

Rastiboroujeni et al. [7], conducted a placebo‐controlled clinical trial to evaluate cosmetic outcomes following rhinoplasty in patients with thick nasal skin treated with topical tretinoin gel. Forty‐nine participants took part in this study, yielding significant findings. It was also found that topical application of tretinoin gel effectively reduces acne in patients with thick nasal skin following surgery. This treatment promoted increased patient satisfaction during the first few months after the procedure. However, the final cosmetic outcomes did not significantly influence it statistically [7].

In the review article by Afzalzadeh et al. [5], it is stated that the use of isotretinoin in rhinoplasty is notoriously associated with the potential for significant improvement in surgical outcomes, especially in individuals with thick nasal skin and sebaceous hyperactivity. Optimal efficacy of this treatment at low doses is demonstrated over a minimum duration of 4 months. Nevertheless, owing to possible toxic side effects of isotretinoin, including teratogenicity, dermatologists must follow protocols and monitor patients carefully [5].

### 4.4 Psychological Aspects

Plastic surgery of the nose, rhinoplasty, and cosmetic surgery, which individuals who would like to change the anatomy of their nose perform, cannot concern themselves with physical alteration only, and must be associated with psychological and emotional evaluation from a psychological point of view [33]. The impacts of these characteristics on the study were analyzed, and their role as facilitating psychological support in this setting strengthens their argument [34]. This significantly influences patients' self‐esteem, as the findings show that psychological support is necessary to regulate patients' self‐esteem. It has been shown that presurgical psychological therapy leads to the establishment of realistic expectations, thereby decreasing the risk of disappointment postoperation [35].

Ferreira et al. conducted a systematic review that enumerates the benefits and risks of using isotretinoin [36]. They stated that isotretinoin could be a good skin preparation agent but that its continuous use before rhinoplasty could induce a deleterious effect on healing. Therefore, it is implied that suspension is needed before the procedure [37].

Other concerns include the possibility that it may affect blood levels, increasing the risk of hematological changes. As such, hematological monitoring during treatment is recommended regularly. The results suggest that the benefit of isotretinoin in skin preparation for rhinoplasty depends on individual patient characteristics, aesthetic goals, and a risk–benefit assessment. The plastic surgeon, dermatologist, and patient must collaborate to ensure the safe and effective use of isotretinoin and to maximize benefits while minimizing potential harm.

### 4.5 Current Guidelines and Best Practices

Ishii et al. [38], developed a clinical practice guideline that provided evidence‐based recommendations for clinicians involved in rhinoplasty procedures or for candidates for such surgery. This guideline aims to improve patient care, diagnosis, and treatment effectiveness and reduce harmful or meaningless variation in clinical practice. The perioperative management of rhinoplasty is based on a comprehensive preoperative evaluation to enhance the functional and aesthetic results [38]. Assessing the nasal airway is essential to ensure that anything compromising the patient's breathing due to the surgery is avoided, but a cosmetic change is necessary. Additionally, clinicians are advised to conduct psychosocial assessments, especially for BDD, to assess the level of unrealistic expectations that may affect patient satisfaction. Patient education regarding the surgical procedure and anticipated outcomes is considered one of the most important factors for enhancing patient satisfaction and minimizing complications [38].

Regarding care and medication, the guidelines recommend minimal use of perioperative antibiotics and stopping antibiotics after 24 h unless in exceptional cases [39]. This practice helps reduce the risk of infection without increasing antibiotic resistance. Additionally, nasal packing during surgery is generally not advised because the literature shows it is ineffective and causes patient discomfort. There is more evidence supporting the use of steroids in the perioperative period than in standard practice [40]. Steroids can help minimize edema and promote tissue healing, but their use should be tailored to the individual patient's needs [40]. Postoperative evaluations are vital for patient satisfaction with nasal function and appearance and should not be performed earlier than 12 months after surgery [31, 37]. This allows assessment of surgical success and complete recovery. Both standard treatments and isotretinoin require careful attention to patient safety. Communication regarding the potential effects of isotretinoin on wound healing must be clear, and any changes to its use should be coordinated with the surgical schedule. Risk reduction involves advising patients about possible problems and recovery factors. Ultimately, these guidelines highlight the importance of personalized treatment plans. Adjustments may be necessary due to isotretinoin use, existing medical conditions, and individual patient goals to achieve optimal surgical results. An effective rhinoplasty care plan prioritizes patient safety, optimal recovery, and satisfaction with the outcomes.

In addition, Hatami et al. [20], reviewed the importance of communicating with patients about the recognized risks of abnormal wound healing associated with systemic isotretinoin treatment. The authors suggest that, whenever possible, surgical treatment be put off until the effects of the retinoids subside. Given the increased need for extreme caution in patients with darker skin phototypes, this recommendation is crucial for this group.

### 4.6 Aesthetic Benefits and Mechanism of Action

It is well known that isotretinoin can reduce sebaceous gland activity, decrease skin thickness, and improve collagen remodeling. Silveira et al. [14], and Yigit et al. [4], have also conducted studies that confirm that isotretinoin reduces dermal thickness and thus is helpful in rhinoplasty when thick nasal skin often seals nasal tip definition. Isotretinoin thins the skin and reduces oil production, thereby improving long‐term surgical outcomes by enhancing postoperative visibility of nasal structures.

### 4.7 Safety and Considerations

Although isotretinoin offers significant benefits in optimizing rhinoplasty outcomes, especially for patients with thick or sebaceous skin, clinicians must remain vigilant about its potential limitations and side effects. Common adverse events across included studies included mucocutaneous dryness (cheilitis, dry nasal mucosa, ocular dryness) and sporadic mild elevations in liver enzymes or triglycerides, which were generally manageable with dose adjustments or symptomatic therapy. Critically, isotretinoin is strictly contraindicated in pregnant women due to its known teratogenicity; rigorous patient counseling and monitoring programs (e.g., iPLEDGE) are essential to prevent fetal exposure. No severe wound‐healing complications, keloid formation, or abnormal scarring attributable to isotretinoin were reported in the included clinical studies when proper monitoring and patient selection were used.

The effectiveness and safety of isotretinoin may vary depending on racial and ethnic skin types, especially in Fitzpatrick skin types III–V found among Middle Eastern, Asian, African, and Latin American populations with thicker, oilier skin. Such patients might benefit more from isotretinoin but also face specific risks like pigmentary changes and abnormal scarring. Personalized evaluation and careful preoperative counseling are therefore advised. Future studies should categorize participants by skin type and ethnicity to improve treatment guidelines and achieve better, fairer results.

### 4.8 Clinical Implications and Future Research

While our systematic review indicates isotretinoin is a promising perioperative adjunct to rhinoplasty in patients with thick nasal skin, clinicians should weigh the therapy's advantages against its inherent risks, and prefer a tailored approach based on thorough risk–benefit analysis and patient‐specific factors. Nevertheless, RCTs with further large‐scale and long‐term dimensions are needed to refine the ideal isotretinoin dosage and duration for rhinoplasty patients, the best timing (preoperative vs. postoperative initiation), potential contraindications, and long‐term skin remodeling effects [20, 37, 41].

## 5 Conclusion

Isotretinoin can enhance aesthetic outcomes, expedite wound healing, and reduce postoperative edema in selected rhinoplasty patients. Nonetheless, strict attention to contraindications, comprehensive monitoring, and patient education are vital for safe administration. There remains a need for large‐scale randomized trials evaluating long‐term outcomes, stratified by ethnicity and skin type, to establish refined, evidence‐based dosing and procedural protocols.

Additionally, isotretinoin use in patients conferred faster resolution of postoperative edema and better skin elasticity, which are primary indications for improvement over surgical precision and recovery. Isotretinoin was safe despite minor adverse effects such as mucosal dryness and transient cheilitis, provided dosing and monitoring were appropriate. There is a need for patient‐specific treatment plans that take into consideration individual patient risk factors and expectations.

Future research should be directed toward performing large‐scale RCTs to establish the protocols for isotretinoin administration in rhinoplasty, including the optimal dosing, duration, and timing relative to surgery. Moreover, long‐term hormonal effects on nasal skin remodeling and the safety of isotretinoin in different patient populations must be further investigated. Rhinoplasty can be refined even more by refining these strategies, which will further enhance the outcomes, lead to higher patient satisfaction, and, ultimately, a higher surgical success rate.

## Author Contributions

Matin Ghazizadeh and Behrouz Barati: study conception, design, critical revision, and intellectual input. Ali Goljanian Tabrizi and Mohsen Jabbari: literature search, study selection, and quality assessment. Sina Hooshmandi: data extraction and synthesis, and manuscript drafting; All authors: final approval.

## Funding

The authors have nothing to report.

## Ethics Statement

The authors have nothing to report.

## Consent

The authors have nothing to report.

## Conflicts of Interest

The authors declare no conflicts of interest.

## Data Availability

The datasets generated and analyzed during the current study are not publicly available but are available from the corresponding author on reasonable request.

## References

[1] R. Cobo and L. Vitery , “Isotretinoin Use in Thick‐Skinned Rhinoplasty Patients,” Facial Plastic Surgery 32, no. 6 (2016): 656–661. 28033642 10.1055/s-0036-1596045

[2] G. C. Peck , “Rhinoplasty: External Shaving Is Invaluable in Treating Thick, Sebaceous Skin,” Aesthetic Surgery Journal 17, no. 6 (1997): 414. 19328096 10.1016/s1090-820x(97)80061-1

[3] A. A. Akhavan , J. H. Pang , S. D. Morrison , and T. Satterwhite , “Gender Affirming Facial Surgery–Anatomy and Procedures for Facial Masculinization,” Oral and Maxillofacial Surgery Clinics of North America 36, no. 2 (2024): 221–236. 38458858 10.1016/j.coms.2024.01.001

[4] E. Yigit , I. T. Rakici , N. Seden , V. Manav , I. Kaygisiz , and O. Yigit , “The Impact of Isotretinoin Therapy on the Nasal Skin Thickness and Elasticity: An Ultrasonography and Elastography Based Assessment in Relation to Dose and Duration of Therapy,” Aesthetic Plastic Surgery 46, no. 4 (2022): 1760–1770. 34820691 10.1007/s00266-021-02663-z

[5] M. R. Afzalzadeh and A. Alizadeh , “Oral Isotretinoin Treatment in Rhinoplasty: A Review,” World Journal of Plastic Surgery 12, no. 3 (2023): 11–17. 10.61186/wjps.12.3.11 PMC10788103 38226191

[6] R. Cobo , J. G. Camacho , and J. Orrego , “Integrated Management of the Thick‐Skinned Rhinoplasty Patient,” Facial Plastic Surgery 34, no. 1 (2018): 3–8. 29409097 10.1055/s-0037-1617445

[7] H. Rastiboroujeni , M. Bakhshaee , M. R. Afzalzadeh , and Y. Nahidi , “Topical Tretinoin in the Management of Thick‐Skinned Rhinoplasty Patients,” World Journal of Plastic Surgery 13, no. 1 (2024): 50–56. 10.61186/wjps.13.1.50 PMC11088733 38742028

[8] A. A. Sazgar , A. Majlesi , S. Shooshtari , M. Sadeghi , A. K. Sazgar , and A. Amali , “Oral Isotretinoin in the Treatment of Postoperative Edema in Thick‐Skinned Rhinoplasty: A Randomized Placebo‐Controlled Clinical Trial,” Aesthetic Plastic Surgery 43, no. 1 (2019): 189–195. 30288563 10.1007/s00266-018-1252-5

[9] B. Guyuron and M. Lee , “An Effective Algorithm for Management of Noses With Thick Skin,” Aesthetic Plastic Surgery 41, no. 2 (2017): 381–387. 28127662 10.1007/s00266-017-0779-1

[10] D. Moher , A. Liberati , J. Tetzlaff , and D. G. Altman , “Preferred Reporting Items for Systematic Reviews and Meta‐Analyses: The PRISMA Statement,” BMJ (Clinical Research Ed.) 339 (2009): b2535. 10.1136/bmj.b2535 PMC2714657 19622551

[11] B. Baser , S. Dev , and S. Mukhopadhyay , “A Comprehensive Approach to Rhinoplasty for Thick‐Skinned Patients,” Journal of Laryngology and Otology 138, no. 5 (2024): 512–519. 38057968 10.1017/S0022215123002220

[12] S. Gold , S. Horvath , and A. Zaenglein , “Emergency Contraception for Patients Taking Isotretinoin,” JAMA Dermatology 160, no. 5 (2024): 487–488. 38568679 10.1001/jamadermatol.2024.0189

[13] M. Pozzi , C. Fàdel , A. Bolletta , R. Cuomo , and C. W. Roxo , “Ethnic Rhinoplasty: Preliminary Results of Our Technique in the Pursuit of the Harmonious Nose,” Journal of Plastic, Reconstructive and Aesthetic Surgery 87 (2023): 135–146. 10.1016/j.bjps.2023.09.036 37839388

[14] C. S. Silveira , L. Azulay‐Abulafia , E. O. Barcaui , M. M. Silva , and A. C. W. Roxo , “Analysis of the Use of Isotretinoin as an Adjuvant in Rhinoplasty,” International Journal of Dermatology 63, no. 2 (2024): 224–231. 38018283 10.1111/ijd.16924

[15] S. Yahyavi , H. Jahandideh , M. Izadi , H. Paknejad , N. Kordbache , and S. Taherzade , “Analysis of the Effects of Isotretinoin on Rhinoplasty Patients,” Aesthetic Surgery Journal 40, no. 12 (2020): NP657–NP665. 32756944 10.1093/asj/sjaa219

[16] G. Dogan , “Possible Isotretinoin‐Induced Keloids in a Patient With Behçet's Disease,” Clinical and Experimental Dermatology 31, no. 4 (2006): 535–537. 16716157 10.1111/j.1365-2230.2006.02140.x

[17] Handbook AM , Australian Medicines Handbook Pty Ltd (Australian Medicines Handbook Pty Ltd, 2012).

[18] A. Layton , “The Use of Isotretinoin in Acne,” Dermato‐Endocrinology 1, no. 3 (2009): 162–169. 20436884 10.4161/derm.1.3.9364 PMC2835909

[19] D. E. Greydanus , R. Azmeh , M. D. Cabral , C. A. Dickson , and D. R. Patel , “Acne in the First Three Decades of Life: An Update of a Disorder With Profound Implications for All Decades of Life,” Disease‐a‐Month 67, no. 4 (2021): 101103. 33041056 10.1016/j.disamonth.2020.101103

[20] P. Hatami , K. Balighi , H. N. Asl , A. Goodarzi , and Z. Aryanian , “Isotretinoin and Timing of Procedural Interventions: Clinical Implications and Practical Points,” Journal of Cosmetic Dermatology 22, no. 8 (2023): 2146–2149. 37326142 10.1111/jocd.15874

[21] R. Rubenstein , H. H. Roenigk , S. J. Stegman , and C. W. Hanke , “Atypical Keloids After Dermabrasion of Patients Taking Isotretinoin,” Journal of the American Academy of Dermatology 15, no. 2 (1986): 280–285. 3018052 10.1016/s0190-9622(86)70167-9

[22] H. Zachariae , “Delayed Wound Healing and Keloid Formation Following Argon Laser Treatment or Dermabrasion During Isotretinoin Treatment,” British Journal of Dermatology 118, no. 5 (1988): 703–706. 2969261 10.1111/j.1365-2133.1988.tb02574.x

[23] L. Fei , M. Bilal , S. A. Qamar , et al., “Nano‐Remediation Technologies for the Sustainable Mitigation of Persistent Organic Pollutants,” Environmental Research 211 (2022): 113060. 35283076 10.1016/j.envres.2022.113060

[24] D. S. Wishart , Y. D. Feunang , A. C. Guo , et al., “DrugBank 5.0: A Major Update to the DrugBank Database for 2018,” Nucleic Acids Research 46, no. D1 (2018): D1074–D1082. 29126136 10.1093/nar/gkx1037 PMC5753335

[25] “iPLEDGE REMS Committed to Pregnancy Prevention [Internet],” (2021), https://ipledgeprogram.com/#Main.

[26] S. Chu , L. Michelle , C. Ekelem , C. T. Sung , N. Rojek , and N. A. Mesinkovska , “Oral Isotretinoin for the Treatment of Dermatologic Conditions Other Than Acne: A Systematic Review and Discussion of Future Directions,” Archives of Dermatological Research 313, no. 6 (2021): 391–430. 33151346 10.1007/s00403-020-02152-4

[27] L. G. Patrocinio , T. G. Patrocinio , and J. A. Patrocinio , “Approach for Rhinoplasty in African Descendants,” Facial Plastic Surgery Clinics of North America 29, no. 4 (2021): 575–588. 34579839 10.1016/j.fsc.2021.06.008

[28] K. A. McDonald , A. J. Shelley , and A. Alavi , “A Systematic Review on Oral Isotretinoin Therapy and Clinically Observable Wound Healing in Acne Patients,” Journal of Cutaneous Medicine and Surgery 21, no. 4 (2017): 325–333. 28362520 10.1177/1203475417701419

[29] L. K. Spring , A. C. Krakowski , M. Alam , et al., “Isotretinoin and Timing of Procedural Interventions: A Systematic Review With Consensus Recommendations,” JAMA Dermatology 153, no. 8 (2017): 802–809. 28658462 10.1001/jamadermatol.2017.2077

[30] D. L. Larson , N. A. Flugstad , E. O'Connor , K. A. Kluesner , and J. A. Plaza , “Does Systemic Isotretinoin Inhibit Healing in a Porcine Wound Model?,” Aesthetic Surgery Journal 32, no. 8 (2012): 989–998. 23110930 10.1177/1090820X12462383

[31] B. C. Allen and J. S. Rhee , “Complications Associated With Isotretinoin Use After Rhinoplasty,” Aesthetic Plastic Surgery 29, no. 2 (2005): 102–106. 15803349 10.1007/s00266-004-0041-5

[32] K. A. McDonald , A. J. Shelley , T. Pierscianowski , and A. Alavi , “Challenging the Cosmetic Procedural Delay Following Oral Isotretinoin Therapy,” Journal of Cosmetic and Laser Therapy 21, no. 1 (2017): 58–60. 10.1080/14764172.2018.1444775 29488816

[33] R. J. Honigman , K. A. Phillips , and D. J. Castle , “A Review of Psychosocial Outcomes for Patients Seeking Cosmetic Surgery,” Plastic and Reconstructive Surgery 113, no. 4 (2004): 1229–1237. 15083026 10.1097/01.PRS.0000110214.88868.CA PMC1762095

[34] S. G. Guven and Y. Gorgulu , “The Impact of Preoperative Psychological Characteristics on Postoperative Satisfaction and Quality of Life in Patients Undergoing Septoplasty and Inferior Turbinate Ablation Surgery,” European Archives of Oto‐Rhino‐Laryngology 279, no. 8 (2022): 4007–4015. 35122128 10.1007/s00405-022-07286-x

[35] A. Sahraian , M. Janipour , A. Tarjan , Z. Zareizadeh , P. Habibi , and A. Babaei , “Body Dysmorphic and Narcissistic Personality Disorder in Cosmetic Rhinoplasty Candidates,” Aesthetic Plastic Surgery 46, no. 1 (2022): 332–337. 34820690 10.1007/s00266-021-02603-x

[36] T. V. F. Ferreira , A. L. C. Fernandes , and M. P. Espósito , “Importance of Psychological Follow‐Up in Rhinoplasty,” Brazilian Journal of Otorhinolaryngology 91, no. 1 (2025): 101498. 39332083 10.1016/j.bjorl.2024.101498 PMC11470162

[37] L. M. Ferreira , C. T. P. Regis , I. A. M. Lopes , L. A. Massara , M. L. D. O. Neves , and R. C. P. D. Campos , “The Use of Isotretinoin in the Postoperative Period of Rhinoplasty in Patients With Thick Skin,” Revista Brasileira de Cirurgia Plástica (RBCP) – Brazilian Journal of Plastic Sugery 39 (2024): e0781.

[38] L. E. Ishii , T. T. Tollefson , G. J. Basura , et al., “Clinical Practice Guideline: Improving Nasal Form and Function After Rhinoplasty,” Otolaryngology‐Head and Neck Surgery 156 (2017): S1–S30. 10.1177/0194599816683153 28145823

[39] D. W. Bratzler , E. P. Dellinger , K. M. Olsen , et al., “Clinical Practice Guidelines for Antimicrobial Prophylaxis in Surgery,” American Journal of Health‐System Pharmacy 70, no. 3 (2013): 195–283. 23327981 10.2146/ajhp120568

[40] M. M. Liu , A. B. Reidy , S. Saatee , and C. D. Collard , “Perioperative Steroid Management: Approaches Based on Current Evidence,” Anesthesiology 127, no. 1 (2017): 166–172. 28452806 10.1097/ALN.0000000000001659

[41] C. K. Kandathil , M. Rossi‐Meyer , M. Saltychev , and S. P. Most , “Effectiveness of Isotretinoin Administration in Rhinoplasty: A Systematic Review,” Facial Plastic Surgery and Aesthetic Medicine 27 (2025): 387–396. 39836149 10.1089/fpsam.2024.0229

